# Circulation and Chemotaxis of Fetal Hematopoietic Stem Cells

**DOI:** 10.1371/journal.pbio.0020075

**Published:** 2004-03-16

**Authors:** Julie L Christensen, Douglas E Wright, Amy J Wagers, Irving L Weissman

**Affiliations:** **1**Departments of Pathology and Developmental Biology, Stanford University School of MedicineStanford, CaliforniaUnited States of America

## Abstract

The major site of hematopoiesis transitions from the fetal liver to the spleen and bone marrow late in fetal development. To date, experiments have not been performed to evaluate functionally the migration and seeding of hematopoietic stem cells (HSCs) during this period in ontogeny. It has been proposed that developmentally timed waves of HSCs enter the bloodstream only during distinct windows to seed the newly forming hematopoietic organs. Using competitive reconstitution assays to measure HSC activity, we determined the localization of HSCs in the mid-to-late gestation fetus. We found that multilineage reconstituting HSCs are present at low numbers in the blood at all timepoints measured. Seeding of fetal bone marrow and spleen occurred over several days, possibly while stem cell niches formed. In addition, using dual-chamber migration assays, we determined that like bone marrow HSCs, fetal liver HSCs migrate in response to stromal cell-derived factor-1α (SDF-1α); however, unlike bone marrow HSCs, the migratory response of fetal liver HSCs to SDF-1α is greatly increased in the presence of Steel factor (SLF), suggesting an important role for SLF in HSC homing to and seeding of the fetal hematopoietic tissues. Together, these data demonstrate that seeding of fetal organs by fetal liver HSCs does not require large fluxes of HSCs entering the fetal bloodstream, and that HSCs constitutively circulate at low levels during the gestational period from 12 to 17 days postconception. Newly forming hematopoietic tissues are seeded gradually by HSCs, suggesting initial seeding is occurring as hematopoietic niches in the spleen and bone marrow form and become capable of supporting HSC self-renewal. We demonstrate that fetal and adult HSCs exhibit specific differences in chemotactic behavior. While both migrate in response to SDF-1α, fetal HSCs also respond significantly to the cytokine SLF. In addition, the combination of SDF-1α and SLF results in substantially enhanced migration of fetal HSCs, leading to migration of nearly all fetal HSCs in this assay. This finding indicates the importance of the combined effects of SLF and SDF-1α in the migration of fetal HSCs, and is, to our knowledge, the first demonstration of a synergistic effect of two chemoattractive agents on HSCs.

## Abstract 2

New results on the migratory behavior of blood cell precursors in the early embryo might be relevant to bone marrow transplants and other clinical therapies

## Introduction

During fetal development, the primary anatomical concentration of hematopoietic stem cells (HSCs) changes location several times. The migration of blood-borne progenitors is essential for the establishment of hematopoiesis in subsequent hematopoietic tissues (Moore and Metcalf 1970; Johnson and Moore 1975; Weissman et al. 1978; Houssaint 1981; Weissman 2000; Akashi and Weissman 2001). The speculation that this fetal migration process occurs as a series of distinct, timed developmental events, wherein large numbers of fetal HSCs simultaneously enter the bloodstream to seed newly forming hematopoietic organs, arose from observations that a decrease in HSCs and/or hematopoietic progenitor numbers in primary hematopoietic tissues occurs just prior to the seeding of newly forming hematopoietic sites (Morrison et al. 1995; Medvinsky and Dzierzak 1996). Hematopoietic precursor numbers increase in intraembryonic sites such as the aorta–gonad–mesenepheros region (AGM) and yolk sac until 11 days postconception (dpc), then decrease, becoming undetectable by 13 dpc (Moore and Metcalf 1970; Muller et al. 1994; Garcia-Porrero et al. 1995; Sanchez et al. 1996; Godin et al. 1999). This decrease in HSC numbers is hypothesized to result from a wave of multipotent progenitors leaving the AGM (Medvinsky and Dzierzak 1996) or yolk sac (Weissman et al. 1978) to seed the fetal liver on 11 dpc. However this occurs, HSCs increase exponentially in the fetal liver from day 12 until day 15 (Ikuta and Weissman 1992; Morrison et al. 1995) or day 16 (Ema and Nakauchi 2000); then, HSC numbers and activity in the fetal liver decrease, although the fetal liver HSC (FL HSC) population continues to proliferate at an equivalent rate. This decrease in HSC numbers in the fetal liver could result from a mobilization of HSCs from the fetal liver to the spleen and bone marrow (Morrison et al. 1995).

While the mechanisms that influence HSC homing and colonization are not completely understood, several experimental models suggest possible regulatory factors. The homing to and colonization of fetal hematopoietic organs by circulating HSCs likely require homing receptor/addressin interactions in the vascular lumen, followed by chemokine/chemokine receptor interactions, integrin/receptor binding, and growth/survival factors. Homing of lymphocytes and leukocytes has been well documented to involve first homing receptor/vascular addressin interactions, resulting in cell tethering and rolling on blood vessel endothelium. The rolling cells respond to a chemoattractant, produced by endothelial or stromal cells within the tissue, by firm adherence to the vessel wall mediated by integrin/receptor interactions. The adhered cells subsequently traverse the vessel wall, migrating toward the increasing gradient of chemoattractant (Butcher and Weissman 1980; Gallatin et al. 1983; Springer 1990; Campbell et al. 1998). A similar cascade of interactions is likely to govern the migration of immature hematopoietic stem/progenitor cells. Mice born with genetic deficiency of the chemokine stromal cell-derived factor-1α (SDF-1α), or its receptor, CXCR4, fail to establish bone marrow hematopoiesis, although fetal liver hematopoiesis is normal (Nagasawa et al. 1996; Zou et al. 1998; Ara et al. 2003). In addition, bone marrow HSCs (BM HSCs) have been shown to migrate selectively in vitro in response to SDF-1α (Wright et al. 2002). SDF-1α may be important both as a chemoattractant (Imai et al. 1999; Peled et al. 1999) and as an activator of adhesion molecules on HSCs (Peled et al. 2000) and may function in the retention and maintenance of fetal HSCs once they reach the hematopoietic niche (Nagasawa et al. 1998; Tachibana et al. 1998; Zou et al. 1998; Kawabata et al. 1999; Ma et al. 1999; Lataillade et al. 2000).

Correct localization of HSCs throughout ontogeny may also involve other specific interactions with the hematopoietic microenvironment (Schweitzer et al. 1996). A factor that is well-established to be important to the maintenance, survival and proliferation of HSCs is Steel factor (SLF) (Broxmeyer et al. 1991; Metcalf and Nicola 1991; Ikuta and Weissman 1992; Li and Johnson 1994; Keller et al. 1995; Holyoake et al. 1996; Goff et al. 1998; Domen et al. 2000). Homozygous deficiency mutations of the SLF-encoding gene *(Sl)*, normally expressed in hematopoietic stromal cells, or its receptor gene *(W)*, encoding the c-Kit tyrosine kinase, result in profound but incomplete defects in hematopoiesis (Russell 1979). Functional hematopoietic cells from Steel ligand-deficient mice *(Sl/Sl^d^)* can be rescued by transplantation to a wild-type host (McCulloch et al. 1965; Fried et al. 1973; Dexter and Moore 1977; Bernstein et al. 1991; Barker 1997). Interestingly, in the lethal *Sl/Sl* background, FL HSCs double their number daily between 13 and15 dpc (Ikuta and Weissman 1992), indicating that factors other than SLF are responsible for fetal HSC expansion. SLF has also been implicated as a chemotactic factor of human (Kim and Broxmeyer 1998) and mouse (Okumura et al. 1996) hematopoietic progenitor cells.

In order to test the hypothesis that fetal HSC migration is a timed developmental event, we collected blood from embryos ranging in age from 12.5 to 17.5 dpc to use in competitive reconstitution assays to measure long-term reconstituting hematopoietic stem cell (LT-HSC) activity. Our results indicate that mouse fetal HSCs are found constitutively rather than episodically in fetal circulation and are present at low numbers throughout mid-to-late fetal development. We also measured the seeding of the fetal spleen and fetal bone marrow during this period. The seeding of these organs is a gradual process occurring over several days and does not appear to involve a large influx of HSC. Finally, we found that FL HSCs migrate in response to the chemokine SDF-1α and that this response is substantially enhanced in the presence of SLF. The enhanced chemotactic response of HSCs to the combination of SLF and SDF-1α is a property of FL HSCs, but not adult BM HSCs.

## Results

### HSC Are Found Constitutively Circulating in Fetal Blood

**Figure 1:**
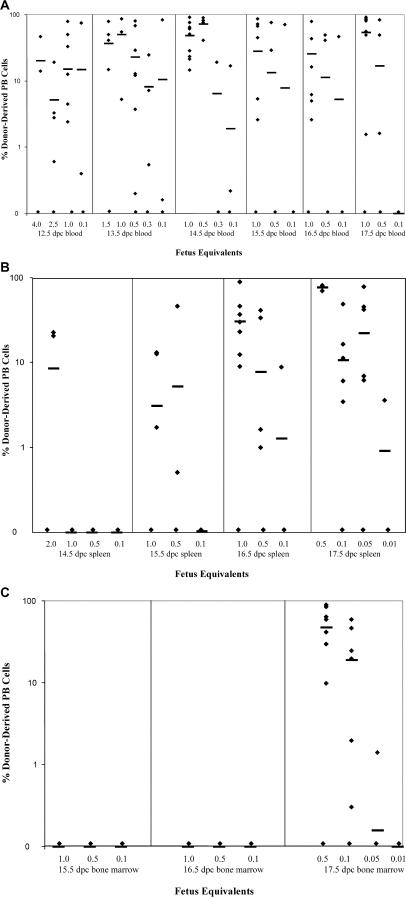
LT-HSC Activity Is Measureable in Fetal Blood, Spleen, and Bone Marrow Cell suspensions of fetal blood (A), spleen (B), and bone marrow (C) were used to competitively reconstitute lethally irradiated recipients. The percentage of donor-derived peripheral blood leukocytes is presented for each dose assayed at 20 wk or more following reconstitution. The bar represents the mean percentage of donor-derived peripheral blood leukocytes in all recipients transplanted with each dose of fetal tissue, blood, spleen, or bone marrow. Fetal tissue from each stage embryo (12.5–17.5 dpc) was transferred in two to three experiments at multiple doses. Positive engraftment was determined by comparison to staining of control mice, which in most cases was less than 0.1%.

**Figure 2:**
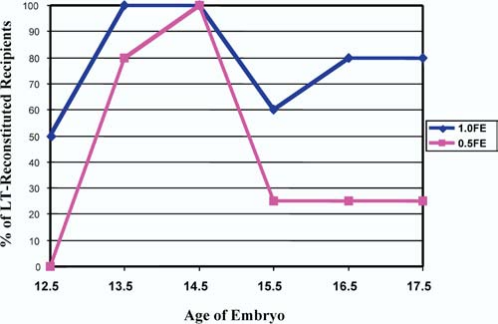
LT-HSC Activity Is Detectable in Fetal Blood (12.5–17.5 dpc) To illustrate an increase in circulating HSCs at 14.5 dpc, the percentage of recipients with donor multilineage reconstitution from fetal blood are plotted for each timepoint (12.5–17.5 dpc), for 1.0 and 0.5 FE.

**Figure 3:**
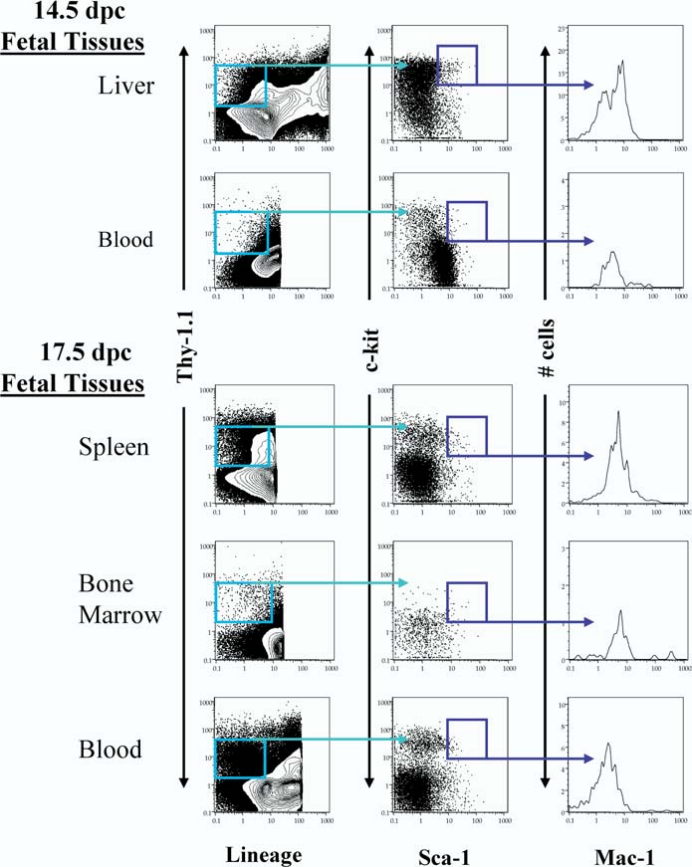
Phenotypic Analysis of HSC in Fetal Blood, Spleen, and Bone Marrow Cells can be identified in fetal circulation, spleen, and bone marrow with the FL HSC phenotype: Lineage^–^ c-Kit **^+^** Sca-1**^+^** Thy-1.1^lo^ Mac-1^lo^. At 14.5 and 17.5 dpc, fetal tissues were analyzed for Lineage^–^ c-Kit **^+^** Sca-1**^+^** Thy-1.1^lo^ and Mac-1^lo^ expression. The leftmost contour plot shows Lineage versus Thy-1.1 staining of live cells. The middle plot shows c-Kit versus Sca-1 staining for gated Lineage^–^ **^/^** ^lo^ Thy-1.1^lo^ cells. The rightmost histogram shows Mac-1 expression by gated Lineage^–^ **^/^** ^lo^ Thy-1.1^lo^ Sca-1**^+^** c-Kit **^+^** cells. Fetal liver, blood, spleen, and BM HSCs have low-level Mac-1 expression. These data are representative of three independent experiments.

To evaluate the presence of rare HSC activity in the fetal circulation, blood was collected from fetuses ranging in age from 12.5 to 17.5 dpc. Blood from a single age group was pooled and assayed by competitive reconstitution. A quantity of fetal cells, measured by fetus equivalents (FEs), was injected into an adult, lethally irradiated congenic recipient, along with a radioprotective dose of host-type bone marrow. A FE was defined as the amount of blood collected from a single fetus of each age group. The amount of fetal blood transplanted ranged from 4 FE to 0.1 FE. The recipient mice were periodically bled and assayed for donor cells of the B, T, and myeloid lineages. Table 1 demonstrates that stem cells capable of long-term multilineage reconstitution (LT-MLR) are found constitutively in the fetal blood throughout the time period assayed, from 12.5 to 17.5 dpc. Figure 1A illustrates the level of donor-derived peripheral blood cells at 20 wk or more. LT-HSC activity is maintained at low but fairly constant levels throughout this time period, with a minor peak apparent at 14.5 dpc (Figure 2). Consistent with these results, cells that display the fetal liver stem cell surface phenotype, c-Kit^+^ Thy-1.1^lo^ Sca-1^+^ Lineage^–^ Mac-1^lo^ (Morrison et al. 1995) can be detected by FACS in fetal circulation at both 14.5 and 17.5 dpc (Figure 3).

**Table 1:**
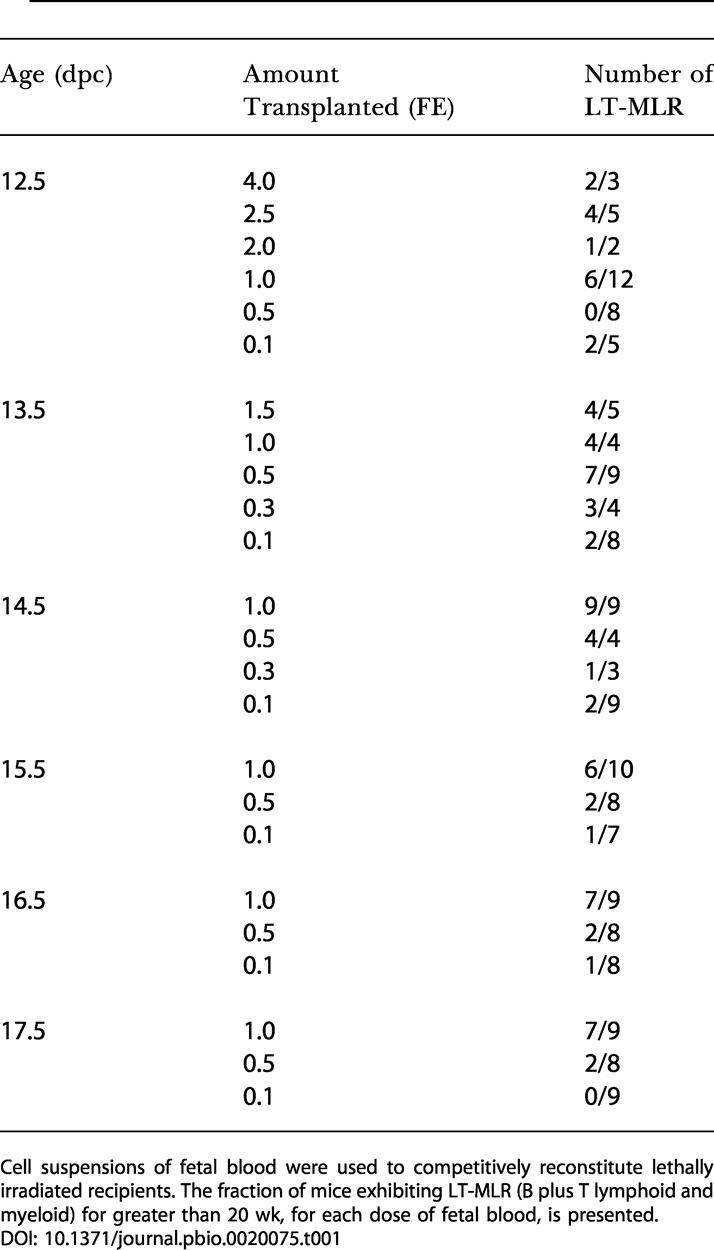
Fetal Blood Titration

Cell suspensions of fetal blood were used to competitively reconstitute lethally irradiated recipients. The fraction of mice exhibiting LT-MLR (B plus T lymphoid and myeloid) for greater than 20 wk, for each dose of fetal blood, is presented

### Seeding of Fetal Spleen and Bone Marrow by HSCs

**Figure 4:**
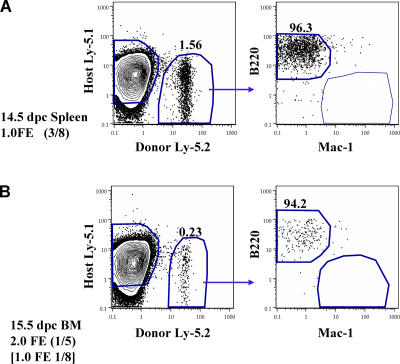
Progenitors Are Found in the Fetal Spleen and Bone Marrow Prior to Colonization by LT-HSC Seeding of the fetal spleen (A) and bone marrow (B) by progenitors unable to provide sustained myelopoiesis precedes colonization of these tissues by HSCs. Reconstituted mice were analyzed for donor contribution in the peripheral blood of B, T, and myeloid lineages at 4 wk post-transplant. Contour plots show gating of donor (Ly-5.2**^+^**) cells and analysis of B220 (B cell) versus Mac-1 (myeloid cell) markers on donor cell populations. At 14.5 dpc, progenitors able to give rise only to B cells were detectable from the fetal spleen in transplantation assays; three of eight recipients receiving 1.0 FE 14.5 dpc fetal spleen cell suspension had donor B cell readout; zero of nine recipients receiving 1.0 FE 14.5 dpc fetal spleen cell suspension LT-MLR. B cell progenitors were detectable from the fetal bone marrow at 15.5 dpc (one of five receiving 2.0 FE) and 16.5 dpc (one of eight receiving 1.0 FE), before detectable HSCs were present. Fetal tissue from each stage embryo (12.5–17.5 dpc) was transferred in two to three experiments at multiple doses. Positive engraftment was determined by comparison to staining of control mice, which in most cases was less than 0.1%.

To measure the kinetics of seeding of the fetal spleen and bone marrow by HSCs, these tissues were similarly assayed by competitive reconstitution for LT-HSC activity. Spleens were collected from 14.5 to 17.5 dpc and fetal bone marrow from 15.5 to 17.5 dpc. For these experiments, a FE was defined as the number of cells collected from a single fetal spleen or bone marrow collected from two femurs plus two tibia. Table 2 illustrates the initiation of long-term multipotent progenitor activity in the fetal spleen and fetal bone marrow. Figures 1B and 1C illustrate the levels of donor-derived peripheral blood cells at 20 wk or more in recipient animals transplanted with fetal spleen or bone marrow cells. Active seeding of the spleen by LT-HSCs occurs at approximately 15 dpc, although infrequent LT-HSCs can be found in 14.5 dpc spleen when multiple embryo equivalents are assayed by transplantation, indicating that very few HSCs initially seed this organ. HSC activity increases daily in the spleen during the period assayed, from 14.5 to 17.5 dpc. While LT-HSC activity is absent from the fetal marrow at the time, it is robustly established in the spleen at 15.5–16.5 dpc. LT-HSC activity can first be detected in the fetal bone marrow at 17.5 dpc. However, when it is established at 17.5 dpc, the initial seeding of the bone marrow is quite robust (see Figure 1C; Table 2), especially given that the amount of bone marrow assayed may only represent approximately 20% of the total bone marrow in the embryo, if the bone marrow is distributed in the fetus as it is in the adult (Smith and Clayton 1970) . The number of spleen HSCs continues to increase as bone marrow colonization proceeds. The appearance of long-term reconstituting activity correlates with visible active erythropoiesis in both tissues (data not shown). HSCs identified by FACS in 17.5 dpc fetal spleen and bone marrow have the FL HSC phenotype, c-Kit^+^ Thy-1.1^lo^ Sca-1^+^ Lineage^–^ Mac-1^lo^ (Morrison et al. 1995; see Figure 3). Within the 14.5 dpc fetal spleen and 15.5 dpc fetal bone marrow are mainly hematopoietic cells that give rise to a burst of B lymphopoiesis but do not provide sustained or detectable myelopoiesis upon transplant to adult recipients (Figure 4). This likely indicates a rapid commitment of HSCs and multipotent progenitors to common lymphoid progenitors or prepro-B cells upon seeding these microenvironments, although it could represent early seeding of these sites selectively by committed progenitor cells rather than HSCs. An alternate, though less likely, explanation is that 14.5 dpc splenic HSCs and 15.5–16.5 dpc BM HSCs are unable to seed the adult environment. In both sites, the likely origin of the immigrant cells is the fetal liver, as HSCs are no longer resident in the yolk sac and AGM at this time (Moore and Metcalf 1970; Muller et al. 1994; Garcia-Porrero et al. 1995; Sanchez et al. 1996; Godin et al. 1999).

**Table 2:**
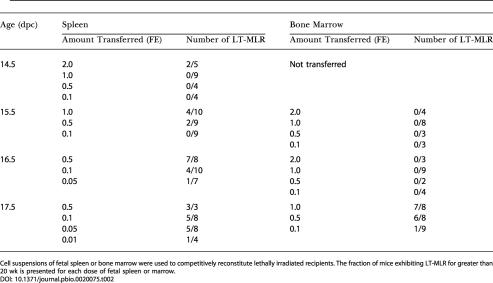
Fetal Spleen and Bone Marrow Titration

Cell suspensions of fetal spleen or bone marrow were used to competitively reconstitute lethally irradiated recipients. The fraction of mice exhibiting LT-MLR for greater than 20 wk is presented for each dose of fetal spleen or marrow

### Synergistic Effects of SDF-1α and SLF on Chemotaxis of Fetal HSCs

**Figure 5:**
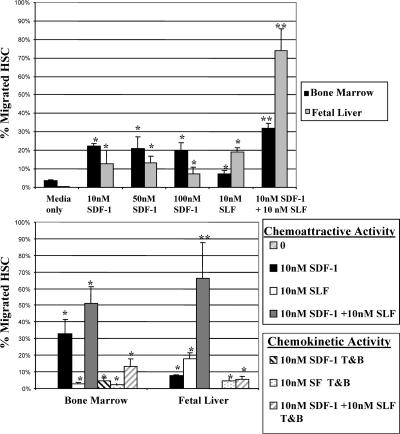
Chemotactic Activity of SDF-1α and SLF on Fetal Liver and Adult BM HSCs HSCs were assayed for their ability to chemotax in a transwell assay in response to the chemokine SDF-1α. Migrating cells were labeled with stem cell markers and analyzed by FACS to determine the actual frequency of migrated HSCs. Like adult BM HSCs, FL HSCs migrate in response to SDF-1α (A), although at reduced levels. The optimal concentration of SDF-1α for both fetal liver and adult BM HSCs was determined to be 10 nM. The migratory effect of SLF was also assayed on FL HSCs and adult BM HSCs. FL HSCs migrate equally well to SLF as SDF-1α, while adult BM HSCs showed a lesser response to SLF. SLF and SDF-1α acted synergistically in their chemoattractive effects on FL HSCs (B). To determine whether migration was due to chemokinetic effects of SDF-1α, SLF, or both, equal concentrations of factors were added to both the top and bottom wells (T&B). Data are presented as the percentage of input HSCs that migrate to the bottom chamber for a representative migration assay, each point was performed in triplicate. These data are representative of three independent experiments. The single asterisk shows a significant increase in percent migration over basal migration (*p* < 0.05). The double asterisk shows a significant increase in percent migration over SDF-1α alone.

To begin to assess whether SDF-1α or SLF may play a direct role in fetal HSC migration, we assayed the ability of FL HSCs to migrate in response to SDF-1α and/or SLF in dual-chamber migration assays. Lineage-depleted fetal liver or adult bone marrow cells were placed in the upper well of a 5-μm transwell chamber, and SDF-1α, SLF, or SDF-1α plus SLF was added to the lower chamber. To evaluate the chemotactic versus chemokinetic effects of SDF-1α and SLF, equal concentrations of factors were added to both the top and bottom chambers. Following a 2 h incubation at 37°C, the cells that had migrated to the lower chamber were collected, stained for HSC markers, and analyzed by FACS to determine the actual number of migrated HSCs. Cell numbers between wells were normalized by the addition of predetermined numbers of fluorescent beads to each well before cell collection (Wright 2002). Like adult BM HSCs, 14.5 dpc FL HSCs migrate in response to a gradient of SDF-1α, albeit at a reduced frequency (Figure 5A). Both FL HSCs and adult BM HSCs displayed an optimal migratory response to SDF-1α at 10 nM. In addition, FL HSCs, but not adult BM HSCs, showed substantial migration, above basal activity, in response to 10 nM SLF. The combination of SLF and SDF-1α had a synergistic affect on the migratory response of FL HSCs, with 70%–90% of the input HSC responding. The increased migration of adult BM HSCs in response to SDF-1α and SLF, above that seen in response to SDF-1α alone, was more modest. Migration in response to SDF-1α of both FL HSCs and adult BM HSCs was largely dependent on the presence of a chemokine gradient (Figure 5B). In contrast, the migration of adult HSCs induced by SDF-1α plus SLF appeared to involve both chemotactic and chemokinetic activity, as inclusion of factors in both the top and bottom of the transwell did not entirely abrogate HSC migration (Figure 5B).

## Discussion

As reported previously, HSCs in the fetal liver double in number daily from 12.5 to14.5 dpc, then decrease in number at 15.5 dpc (Ikuta and Weissman 1992; Morrison et al. 1995). At 12.5 dpc, approximately 1,200 HSCs are present, 2,430 at 13.5 dpc, and 5,100 at 14.5 dpc. However, rather than doubling to 10,200, only 4,350 FL HSCs are found at 15.5 dpc (Morrison et al. 1995). This failure of FL HSCs to continue doubling their numbers from day 14.5 to day 15.5 is unexpected, because the same percentage of HSCs remained as actively in cycle on day 15.5 as on day 14.5 (Morrison et al. 1995). Thus, about 5,800 HSCs are “missing” from the 15.5 dpc liver, possibly because they migrated via the blood to seed the spleen or bone marrow. In order to test this hypothesis quantitatively, we assayed fetal blood, spleen, and bone marrow for HSC activity. However, our results indicate that HSCs are found in the fetal blood at low but fairly constant levels during much of late fetal development. Furthermore, we were unable to find evidence of a large influx of HSCs into the fetal spleen and bone marrow at this timepoint. The seeding of the fetal spleen and bone marrow is progressive and does not appear to be the result of a large, developmentally timed migration. Thus, the decrease in FL HSC numbers at 15.5 dpc is more likely explained by their differentiation out of the HSC pool; this may be due to the onset of hepatic differentiation in the fetal liver and an induction of hepatic growth factors (Kinoshita et al. 1999). Initiation of differentiation of HSCs to multipotent and oligopotent progenitors at 15 dpc may account for the increase in colony-forming unit–spleen (CFU-S) activity (a mixture of HSCs, multipotent progenitors, common myeloid progenitors, and megakaryocyte/erythrocyte-restricted progenitors [Na Nakorn et al. 2002]) in the fetal liver at 17 dpc, observed in other studies (Niewisch et al. 1970; Wolf et al. 1995) at this and later timepoints, that occurs despite the decreasing number of HSCs in the liver following 15.5 dpc (Morrison et al. 1995; Ema and Nakauchi 2000). HSCs are found in the blood after the establishment of active circulation at 9 dpc (Toles et al. 1989) and are present in the fetal blood at all times following the onset of circulation. As reported by Kumaravelu et al. (2002), we see an increase in circulating HSCs between 12 and 13 dpc. In adults, the residence time of HSCs in the blood is about 1–3 min (Wright et al. 2001). If this is likewise true in fetuses, then long-term reconstitution is likely an accurate measure of HSC flux during fetal life. Alternatively, if the transit time of blood-borne HSCs decreases at 15 dpc and migrating HSCs lose long-term repopulating potential upon initial seeding of the spleen, we may have been unable to measure such a developmentally timed migration.

**Figure 6:**
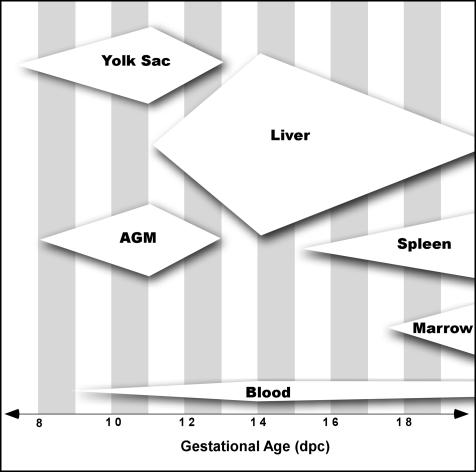
An Updated Model Illustrating the Location and Relative Frequencies of Fetal HSCs in the Embryo HSCs are found constitutively at low numbers in fetal blood following the onset of circulation. Seeding of developing hematopoietic tissues by long-term HSCs is gradual and is not due to a large influx of cells. The large decline in HSC numbers seen in the fetal liver following 14 dpc is most likely the result of differentiation signaled by the developing hepatic environment rather than a timed migration to the fetal spleen and bone marrow.

As measured by reconstitution, a slight peak of circulating embryonic HSCs appears at 14.5 dpc. The slight decrease of circulating HSCs at 15.5 dpc could also be due to HSCs leaving the circulation to seed the spleen and later the bone marrow. The fetal spleen and bone marrow are initially seeded by progenitors unable to provide detectable or sustained myelopoiesis, indicating additional requirements not found in these immature hematopoietic tissues are needed for LT-HSC seeding, maintenance, or both. Our data indicate that fetal liver-derived HSCs (c-Kit^+^ Thy-1.1^lo^ Lineage^–^ Sca-1^+^ Mac-1^lo^) begin to seed the fetal spleen and bone marrow on 14.5–15 dpc and 17.5 dpc, respectively. We propose that fetal HSCs are continuously entering circulation and functionally engraft specialized stem cell niches as they develop (Figure 6).

Our results stand in contrast with those previously reported by Delassus and Cumano (1996) and Wolber et al. (2002). Using an in vitro differentiation system, Delassus and Cumano (1996) reported that blood multipotent activity is present in the fetal blood from 10 dpc to 12 dpc and then becomes undetectable. Likewise, we were unable to detect a decrease in circulating HSCs between 13–15 dpc, as reported by Wolber et al (2002), measured by CFU-S. We utilized a competitive reconstitution assay, which is the most stringent and reliable indicator of LT-HSC activity to assay functionally for LT-HSCs in fetal circulation. We were able to detect circulating HSCs throughout the period measured, 12.5–17.5 dpc.

Studies indicate that HSC trafficking to and retention in the bone marrow relies on the chemokine SDF-1α and its receptor CXCR4 (Aiuti et al. 1997; Dutt et al. 1998; Kim and Broxmeyer 1998; Kawabata et al. 1999; Ma et al. 1999; Peled et al. 1999; Ara et al. 2003). We have previously shown that BM HSCs migrate only in response to SDF-1α, a large panel of chemokines (Wright et al. 2002). However, a recent study indicated that 11 dpc c-Kit**^+^** fetal liver hematopoietic progenitor cells respond poorly to SDF-1α (Mazo et al. 2002). The discrepancy between our results and those of Mazo et al. may relate to differences in the developmental stage of the cells examined or to differences between HSCs and progenitors. At 11 dpc, the fetal liver is beginning to be seeded, and based on knockout studies, SDF-1α is not required for seeding of the fetal liver (Kawabata et al. 1999). In contrast, our study demonstrates clearly that 14.5 dpc FL HSCs do migrate in response to SDF-1α, although at a reduced frequency as compared to adult BM HSC chemotaxis to SDF-1α.

We also found that the migratory response of fetal HSCs to SLF was equal to the response to SDF-1α. The migratory response of fetal HSCs to SDF-1α in combination with SLF was synergistic. This finding indicates the importance of the synergistic effects of SLF and SDF-1α in the migration of fetal HSCs. In contrast, SLF alone or in combination with SDF-1α did not evoke a greatly enhanced migratory response from adult BM HSCs. At best, the combination of SLF and SDF-1α had additive affects on the chemoattractive response of adult BM HSCs. A synergistic effect of two chemoactive agents has not, to our knowledge, been directly demonstrated before for HSCs. The substantial migratory response of 14.5 dpc FL HSCs to the combination of SLF and SDF-1α could explain the increased severity of the Steel mutant phenotype for bone marrow versus fetal liver hematopoiesis (Ikuta and Weissman 1992). The late embryonic lethality seen in the *Sl/Sl* mutant may be due to the inability of FL HSCs to efficiently seed the bone marrow. The chemotactic response to these factors by 14.5 dpc fetal HSC may also underlie the superior reconstituting ability of FL HSCs (Morrison et al. 1995; Rebel et al. 1996), as SLF is released by bone marrow cells following cytoreductive injury and thus may enhance recruitment of infused FL HSCs over adult BM HSCs, which do not respond to SLF.

Our lab has recently described a physiological process in which low numbers of BM HSCs rapidly but constitutively traverse the bloodstream of normal mice to seed unoccupied bone marrow niches (Wright et al. 2001). We now propose that this process, which may serve to survey barren niches in adult bone marrow, also functions to seed newly forming fetal hematopoietic tissues as suitable microenvironments develop. Protocols that induce mobilization of HSCs for clinical hematopoietic cell transplantation may mimic mechanisms, already in place, that allow naturally occurring migration and engraftment of HSCs in the fetus and adult. Further investigation into the mechanisms that regulate these naturally occurring migrations may yield both an improved understanding of the importance of HSC migrations for hematopoietic development and improved protocols for clinical bone marrow transplants.

## Materials and Methods

### 

#### Mouse strains

The C57BL/Ka-Thy-1.1/Ly-5.2 (Thy-1.1, Ly-5.2) donor and C57BL/Ka-Thy-1.2 (Thy-1.2, Ly-5.1) recipient mouse strains were bred and maintained at the Stanford University Laboratory Animal Facility, Stanford, California, United States. All mice were routinely maintained on acidified water (pH 2.5). Irradiated recipient mice were more than 8 wk old at the time of irradiation. All protocols were approved by the Administrative Panel on Laboratory Animal Care at Stanford University School of Medicine.

#### Antibodies

A20.1 (anti-Ly-5.1, CD45.2, FITC-conjugated; BD Biosciences [Pharmingen], Palo Alto, California, United States) and AL1-4A2 (anti-Ly-5.2, CD45.1, Texas Red conjugate) were used to analyze donor and host cells following reconstitution. Blood analysis included 6B2 (anti-B220), KT31.1 (anti-CD3), GK1.5 (anti-CD4), 53-6.7 (anti-CD8), 8C5 (anti-Gr-1), and M1/70 (anti-Mac-1).

The monoclonal antibodies used in immunofluorescence staining for HSC analysis included 2B8 (anti-c-Kit, APC conjugate), 19XE5 (anti-Thy-1.1, FITC conjugate), E13 (anti-Sca-1, Ly6A/E, Texas Red conjugate), and M1/70 (anti-Mac-1, PE conjugate). Lineage marker antibodies included 6B2 (anti-B220), KT31.1 (anti-CD3), GK1.5 (anti-CD4), 53-7.3 (anti-CD5), 53-6.7 (anti-CD8), Ter119 (anti-erythrocyte-specific antigen), 8C5 (anti-Gr-1), and M1/70 (anti-Mac-1). The antibodies were purified and conjugated within our lab. Each antibody was titrated and used at predetermined optimal concentrations (highest signal with lowest background following staining of control spleen or bone marrow cells).

#### Fetal Tissue Preparation

Timed pregnancies of C57BL/Ka-Thy-1.1/Ly-5.2 mice were used to obtain embryos. The day the vaginal plug was observed was designated as 0.5 dpc. The uterus was removed and washed to remove maternal blood. Fetuses were carefully removed to prevent contamination with maternal blood. Fetuses were then decapitated in Hanks' balanced salt solution containing 5 mM EDTA and allowed to bleed out. Fetuses were passaged through several dishes of media until completely pale. Blood was combined and centrifuged. Blood was either prepared for injection by sedimenting RBCs in dextran followed by lysis of erythrocytes in 0.15 M ammonium chloride, 0.01 M potassium bicarbonate solution on ice, or remained unmanipulated and was injected directly into recipients. Results from both preparations were comparable. Spleens were obtained by first removing the spleen and stomach to a dish of Hanks' balanced salt solution containing 2% FBS. The spleens were then peeled from the surface of the stomach and placed in a clean dish of media. Femurs and tibia were removed and cleaned of muscle tissue. Spleens and bone were dissociated using the rubber end of a 1 ml-syringe plunger and filtered through nylon mesh. Blood and tissues were collected from fetuses obtained from at least three pregnant females for each timepoint measured. Fetuses that appeared developmentally advanced or delayed in any age group were discarded.

#### Competitive reconstitution

Adult recipient mice were lethally irradiated with a split dose of 950 rad as previously described (Morrison and Weissman 1994). Recipient mice were anesthetized with 3% isoflurane. Fetal cells were transferred by retroorbital injection along with a radioprotective dose of 3 × 10^5^ CD45 congenic (recipient-type) whole bone marrow cells. Recipient mice were periodically bled to analyze peripheral blood for donor B and T lymphocytes and myeloid cells. Recipients were determined to have LT-MLR if all three of these donor subsets were present for greater than 20 wk. Positive engraftment was determined by comparison to control mice, and in most cases the threshold for positivity was less than 0.1%.

#### Chemotaxis assays

FL HSCs and adult BM HSCs were prepared as for antibody staining. Cells were stained with a lineage cocktail of the same purified rat IgG monoclonal antibodies used for FACS sorting. The cells were then depleted by magnetic selection using anti-rat IgG beads as per manufacturer's instructions (Dynal Biotech, Oslo, Norway), followed by a 1h incubation in RPMI media (GIBCO-BRL, San Diego, California, United States) containing 10% FBS in a tissue culture flask at 37°C to remove adherent cells. Dual-chamber chemotaxis assays were performed using 24-well plates with 5-μm pore size inserts (Costar/Corning, Corning, New York, United States), as previously described (Wright et al. 2002). SDF-1α-containing medium (PeproTech, Rocky Hill, New Jersey, United States) or SLF-containing medium (R & D Systems, Minneapolis, Minnesota, United States) was added to the lower chamber, and 100 μl of a cell suspension (5 × 10^6^ or 1 × 10^7^ cells/ml) of lineage-depleted cells was placed in the upper chamber. To measure chemokinetic movement, factors were also added to the upper chamber at the same concentration as the lower chamber. Following a 2 h incubation, the upper chamber was removed. A known quantity of fluorescent beads was added to the lower chamber for normalization of migrated HSCs. Migrated cells were removed from the lower chamber and stained with PE-conjugated antirat IgG. After washing, cells were incubated with rat IgG and then labeled with directly conjugated lineage PE, c-Kit APC, Sca-1 Texas Red and Thy-1.1 FITC antibodies. Cells were analyzed by FACS to enumerate migrated HSCs.

#### Statistics

Results shown in Figure 5 represent the mean plus the standard deviation. Significant differences were determined using a two-tailed Student's *t*-test. A *p* value of <0.05 was considered significant.

## Supporting Information

### 

#### Accession Numbers

The LocusLink (www.ncbi.nlm.nih.gov/LocusLink/) accession numbers of the gene products discussed in this paper are CXCR4 (LocusLink ID 12767), SDF-1α (LocusLink ID 20315), and SLF (LocusLink ID 17311).

## Abbreviations

AGM: aorta–gonad–mesenepheros region
BM HSC: bone marrow hematopoietic stem cell
CFU-S: colony-forming unit–spleen
dpc: days postconception
FE: fetus equivalent
FL HSC: fetal liver hematopoietic stem cell
HSC: hematopoietic stem cell
LT-HSC: long-term reconstituting hematopoietic stem cell
LT-MLR: long-term multilineage reconstitution
SDF-1α: stromal cell-derived factor-1α
SLF: Steel factor
